# Challenging Operations: An Ethical Framework to Assist Humanitarian Aid Workers in their Decision-making Processes

**DOI:** 10.1371/currents.dis.96bec99f13800a8059bb5b5a82028bbf

**Published:** 2014-06-23

**Authors:** Caroline Clarinval, Nikola Biller-Andorno

**Affiliations:** Institute of Biomedical Ethics, University of Zurich, Zurich, Switzerland; Institute of Biomedical Ethics, University of Zurich, Zurich, Switzerland; Division of Medical Ethics, Harvard Medical School, Boston, Massachusetts, USA

**Keywords:** decision-making, disasters, Ethical framework, humanitarian aid workers

## Abstract

Introduction: This paper aims to raise awareness regarding ethical issues in the context of humanitarian action, and to offer a framework for systematically and effectively addressing such issues. Methods: Several cases highlight ethical issues that humanitarian aid workers are confronted with at different levels over the course of their deployments. The first case discusses a situation at a macro-level concerning decisions being made at the headquarters of a humanitarian organization. The second case looks at meso-level issues that need to be solved at a country or regional level. The third case proposes an ethical dilemma at the micro-level of the individual patient-provider relationship. Discussion: These real-life cases have been selected to illustrate the ethical dimension of conflicts within the context of humanitarian action that might remain unrecognized in everyday practice. In addition, we propose an ethical framework to assist humanitarian aid workers in their decision-making process. The framework draws on the principles and values that guide humanitarian action and public health ethics more generally. Beyond identifying substantive core values, the framework also includes a ten-step process modelled on tools used in the clinical setting that promotes a transparent and clear decision-making process and improves the monitoring and evaluation of aid interventions. Finally, we recommend organizational measures to implement the framework effectively. Conclusion: This paper uses a combination of public health/clinical ethics concepts and practices and applies them to the decision-making challenges encountered in relief operations in the humanitarian aid context.

## Introduction

In the field of humanitarian action, making ethically justified decisions is challenging. In disaster affected contexts the needs faced by populations oftentimes outweigh personal and material resources^1^. AT the same time, well-intentioned humanitarian actors focus on meeting those needs. While addressing those needs, these actors promote values such as independence, impartiality, saving lives, prevention and preparedness, providing aid, promoting health and well-being^2^^-^ 4 ^,^ 26.

In many cases, these values cannot simply be implemented, but need to be specified and possibly weighed against each other. For example, in contexts where a value such as saving lives conflicts with personal security, aid workers may choose to refrain from engaging in assisting distressed populations due to the lack of security on the ground 5 . Further, Calain^6^ emphasizes that “humanitarian principles are often misrepresented as primary sources of legitimacy.” Doing “the right thing” requires more than good intentions; it also asks for careful consideration and an explicit, systematic approach. In this paper, we propose a framework of principles, procedural steps, and organizational resources to ensure that the decision-making process is consistent and fair, and that humanitarian actors can work through these issues in a well-argued and efficient manner.

Dilemmas arise frequently (but not exclusively) in the context of resource allocation, particularly since humanitarian aid is a limited resource, with programmatic decisions being based on unmet needs^7^. Distribution occurs according to parameters such as the role of other actors, prioritization across contexts of available resources, and the cost and benefits to an organization of specific interventions. Moreover, the organization’s mandate, responsibility, expertise, capacity, impact, and foreign and domestic policy are involved 7 ^,^ 8. The multitude of potentially conflicting factors – some of ethical or strategic relevance - demonstrates the necessity of a framework for working through the process of clarifying, specifying, and weighing these factors in an accountable way.

In usual practice, the humanitarian discourse neither disentangles conflicting values nor identifies and clearly names ethical issues. Rather, ethical problems tend to be reduced to matters of geopolitics or management. For example, assisting populations in contexts with limited access must consider the critical trade-off between the principles of beneficence and justice. Due to the lack of security on the ground, agencies are unable to confirm whether all of the goods reach the population successfully. As long as this process of ethical decision-making is neglected, operational decisions might be made in a discriminatory way, potentially skewing assessments and affecting programmatic outcomes.

One reason for this situation may be that managers in charge of humanitarian operations are unlikely to be trained in ethics. Hence, ethical issues remain unidentified and therefore unaddressed. Fink and Redaelli^9^argue that donor governments are biased in funding aid operations that are geographically closer, politically compatible and in oil exporting countries. Results of their study further show that political factors are as important as the prevailing needs.

Overall, the resource allocation processes seem to lack structure and transparency, hindering a comprehensive evaluation of the impact of aid. Therefore, decision-making processes of aid agencies are stifled and run the risk of adding further damage to their beneficiaries.

This paper aims to meet this challenge which hinders the effectiveness of humanitarian efforts. First, we use three cases situated at macro-, meso- and micro-levels to illustrate different ethical conflicts that may arise in the context of humanitarian aid. Second, we propose a novel ethical framework suggesting a set of normative values that might be considered by humanitarian aid workers during their decision-making process. This set of values draws parallels to certain public health ethics frameworks, since humanitarian aid workers often focus on assisting large populations. Third, to address those processes, we propose a ten-step approach that is based on clinical ethics models. As such, tools that have already proven useful for addressing dilemmas in the clinical setting are applied to a humanitarian aid context for the first time. Finally, we propose certain organizational measures that aim to support humanitarian aid organizations in their effort of implementing the framework and securing well-argued and fair decision-making in their daily work.

## Cases

The following cases^[1]^ have been chosen to illustrate the ethical dimension of many decisions in humanitarian aid. They reflect instances that have occurred in practice, and are likely to describe situations that are familiar in some form or other by those active in the field.

**1.1. Macro-level (headquarters): Repeated budget allocation – a matter of fair process**

For most humanitarian organizations, the management of operational budgets rely on suggested interventions from the field and the amount of financial resources sourced by fundraisers. Humanitarian aid has restricted capacities, which usually leads to a prioritization of populations in acute crisis. In these situations, emergency appeals can be launched to raise additional funds. However, the allocation of aid to more chronic contexts in the state of deterioration, stabilization, or imminent recovery also needs scrutiny, because the respective populations might be over- or underserved. The budget of these more long-term operations is usually annually reviewed and adjusted.

Case: Organization X holds a budget committee meeting at headquarters. The available budget is clearly insufficient to cover all the proposed interventions; in fact, it is ten percent smaller than last year. Apart from a few new interventions, most of programs have run for several years. Some programs were implemented very successfully, but as those working in the field argue, they must receive continued funding in order to secure sustainable improvement. Another program has had poor outcomes but is situated in an area of particular strategic importance for the organization, generating a great level of interest among the media and public. Another has done poorly, but is working under difficult circumstances and provides crucial support to a severely deprived population. How should the committee allocate the budget? Should they reduce the funding of all projects evenly? Should some projects be treated preferentially, while others are downsized or discontinued? If so, what criteria should be used in making the decisions: urgency, need, population size, location, success of the intervention, political considerations, or still other factors?

As illustrated in this case, repeated budget cuts in practice are likely to be carried out at central headquarter level. If decisions are made in a top-down fashion behind closed doors, the expert staff in the concerned countries are unlikely to participate in the decision-making process, leading to a lack of transparency and accountability. Populations affected by disasters are subjected to arbitrary budget allocations and may or may not benefit from external aid, depending on who voices their concerns most effectively. Framing budget allocation issues is a matter of justice rather than merely a technical matter of finance. It also allows the use of criteria for fair process, such as transparency, moral relevance of the arguments justifying the decision, and allowing the the decision to be revised in light of new information^10^.

**1.2. Meso-level (country/region): Aid workers - mere means instead of ends in themselves?**

Humanitarian resources are allocated on the basis of both political grounds and need^9^. Given this mix of underlying motivations, the declared purpose of interventions is likely to become obscured. For example, one-off food distributions involve times when food rations are allocated to certain populations over a short period of one or two months. During this period, it is unlikely that the nutritional status of the targeted populations will improve significantly, because effective food aid requires a prolonged intervention, supported by surveillance. This has increasing relevance in areas where the populations are food insecure. However, food aid is also provided in areas where populations are not necessarily food insecure. Therefore, food assistance does not always meet the purpose of improving a population’s nutritional status but has other aims, such as improving access to certain local groups like leaders, political factions, or rebels.

Case: In a war-stricken area, a humanitarian aid organization carries out a one-off food distribution that is unlikely to significantly improve the nutritional status of a given population. The primary aim is to establish contact with local leaders in order to improve the organization’s acceptability. The staff deployed to this highly volatile context remain unaware of the underlying operational reasons and believe that the distributions would serve the purpose of improving the nutritional status of a food-insecure population. When fighting begins between rebel groups near the distribution site, aid workers decide to abandon the distribution. They request a discussion with program leaders about the mission’s purpose. When learning the real goal of the intervention, they feel deceived and abused, and some state they would not have taken the risks just for “the glory of the organization.” 

A justification for this intervention could have been that in the context of armed conflict, involving all parties in the conflict is acknowledged as paramount to promoting acceptability of the humanitarian organization and its staff, which will in turn contribute to their safety and security. Since this was not communicated from the outset, the staff on the ground rightly felt that they had been used for a purpose that they were not informed about and had not consented to. As a result, they lost trust in their organization and the self-serving agenda which would merely enhance the organization's reputation or ensure that the area was not left to competitors. Aid workers must be in a position to make an informed personal decision whether the goals of the mission justifies the risks involved. Omitting this step disrespects the autonomy of individuals who are exposed to potential harm.

**1.3. Micro-level (delegate/beneficiary level): the cost of doing good – the boundaries of beneficence**

Humanitarian organizations are regularly involved in the capacity-building programs of local health care providers. In such programs, expatriate physicians or other healthcare professionals train and build the capacity of local providers, which requires meeting a certain set of preconditions. For instance, the roles and responsibilities of the expatriate in charge and the local physicians need to be clarified at the outset^11^. The expatriate physician’s role is to ensure that the conditions for knowledge transfer are optimal and that the treatment benefits the patient.

Case: A humanitarian organization wishes to remain present in a specific context and opts for a capacity-building program for surgeons. An expatriate physician is tasked to train his local counterparts. During one surgical procedure, it is agreed that the physician will teach the local surgeons how to set an external fixator to treat a complex fracture. However, the local physicians do not follow the instructions of the physician in charge, which results in harm to the patient, eventually leading to the amputation of a limb. From the expatriate surgeon’s view, the reasons for this are mainly linked to unresolved matters of power and status, rendered more complex through the intercultural setting. Subsequent post-operative care is exclusively provided by the local surgeons, as the expatriate surgeon lacks access to the facilities on a regular basis. No follow-up of the patient is possible by the physician who should act as trainer. The incident is reported to the organization’s managers, who agree that no further steps are needed to address the situation, accepting the harm as a kind of “collateral damage” for an otherwise useful program.

For the organization, the emphasis was on ensuring a continued presence in the country, accepting a certain degree of harm. However, it cannot simply be assumed that high-risk actions potentially resulting in significant harm to patients are justified for the sake of remaining present in certain contexts, at least if this means pursuing a political rather than a purely humanitarian goal. Programs that deploy highly qualified staff such as surgeons and have the goal of implementing projects bearing high risks to the affected population need to be thoroughly evaluated in regard to potential harms and benefits, as well as their distribution. At present, the decision-making structures do not explicitly insist on carrying out an in-depth review of the benefits or risks that certain operations have on the beneficiaries. Ethical aspects should be identified before deploying teams or at least as dilemmas arise. Programs should also be regularly monitored and evaluated in order to avoid causing unnecessary and unjustified harm.

These three cases illustrate that humanitarian contexts are riddled with ethical issues on every level and they vary in complexity. Providing a simple, straightforward answer to these issues is difficult and often impossible. First, there appears to be a gap in the literature regarding the identification of possible ethical issues and their distinction from geopolitical or strategic questions. Second, there may be genuine conflicts of principles or values that cannot be solved by decreeing a single “solution” but need to be resolved by identifying and critically examining arguments in light of input from various perspectives and the relevant empirical data available. For instance, deciding who should receive limited relief goods requires weighing ethical principles such as beneficence and non-maleficence, or equality and needs. This process would benefit from an ethical framework and standardized steps tailored to the humanitarian context that are currently unavailable. In absence of set criteria on how to weigh the principles, we suggest pointing towards Beauchamp and Childresses’^22^ process and conditions of weighing and balancing principles highlighting that ‘balancing is the process of finding reasons to support beliefs about which moral norms should prevail.’^[2]^

## Ethical framework

Generally, the decision-making processes involved in allocating and delivering limited humanitarian resources strive to improve people’s well-being. However, decision-making processes and the degree to which ethical issues are identified and addressed differ across organizations. No structured reference framework currently exists that can assist aid workers in identifying potential ethical issues and support them in their decision-making process.

In this section, we propose such a framework, drawing on resources that have been developed in the fields of public health and clinical ethics. In public health ethics, the focus is on eventual dilemmas arising between individual rights and community benefit^12^. Such dilemmas may arise in the humanitarian context, since programs aim to assist populations in a way that might affect individual rights. On the other hand, we refer to processes that have proven useful in the clinical context, with clinical ethics offering guidance on the management and resolution of moral conflicts arising in-patient care^13^. Likewise, humanitarian aid workers care for individuals and populations affected by disasters and may therefore be confronted with similar issues.

Distilling relevant experiences and insights from both public health ethics and clinical ethics and adjusting them to the context of humanitarian aid may allow the young field of ethics of humanitarian action to advance in an efficient way, while benefitting from achievements in neighboring fields.

**2.1. Public health ethics frameworks**

In public health, a number of ethical frameworks have been proposed 14. They highlight values such as promoting respect for people while protecting public health, producing maximal benefits while minimizing harm, cost-effectiveness, and social justice. Similarly, one could argue that the distribution of humanitarian relief relies on similar values, insofar as they focus on large populations. For example, the allocation of food assistance programs may target several camps, each hosting hundreds of thousands of internally displaced persons or refugees. As such, drawing parallels to current public health ethics frameworks is reasonable when responding to the needs caused by disasters.

A challenge in addressing ethical issues in public health is that much policy-making remains in silos, which hinders the joint effort at forming cross-sectoral public policy^15^. A similar case is seen in humanitarian action. Programs appear to be distinguished by disciplines such as civil engineering, medicine, agronomy, or law. They might also be differentiated into departments such as water and habitat interventions, health interventions, or food and non-food interventions. Changing this would require altering the structure of aid organizations. Meanwhile, the dilemma that arises concerns “the rights and freedoms of individuals, and the needs and good of the community”^12^, which cannot remain unaddressed in the field of humanitarian action and requires the development of certain skills and tools.

**2.2. Clinical ethics frameworks**

In referring to clinical ethics frameworks in the context of humanitarian action, we acknowledge that humanitarian action evolves in a complex environment involving multiple relationships. Some of these are hierarchical, and some are fraught with cross-cultural issues. Deciding the correct course of action can be a formidable challenge under such circumstances. Yet, decisions must be made in clinical settings according to professional norms^13^ and in humanitarian action, these decisions sometimes need to be made quickly and with consideration of their potentially very serious impact. Although clinical practice is mainly concerned with individuals while the focus of humanitarian aid is on populations, both perspectives converge in the need to respond to challenging situations in an efficient and clearly-argued manner. Having a defined procedural process can improve the transparency, accountability, and quality of decisions.

In the context of clinical settings, complex decisions, including the allocation of limited resources, have to be made frequently. Clinical ethicists have developed various tools that assist decision-makers in this difficult task. Key features of some of these tools^[3]^ are:

1. Situation analysis involving all concerned actors
2. Description of the key values at stake and their potential conflict
3. Critical examination of the arguments at stake
4. Description of options and their potential and forecasted consequences on each actor
5. Weighing of options
6. Agreeing on the way forward

In a suitably adapted form, similarly structured multi-step approaches might also support the decision-making processes in humanitarian programs. In addition, clinical ethics has developed models for fostering expertise in clinical institutions without moral responsibility being delegated to an “ethics expert” (see “Institutional requirements” below).

**2.3. Identifying ethical issues and making decisions in humanitarian aid**

Our suggested ethical framework is an attempt to engage the humanitarian community in the discourse of identifying ethical issues in the context of humanitarian action and to provide tools to strengthen the decision-making process of humanitarian aid workers. The framework draws from previously developed concepts that have proven useful in public health and clinical ethics. This framework has three main aims.

First, it intends to emphazise that humanitarian action is based on *sets of values *referred to explicitly or implicitly by humanitarian actors, organizations and donors^25^ . Stakeholders or agencies may embrace different sets of values^16^. Certain values represent the substantive views of the organization such as impartiality, independence or religious beliefs. Others values mirror procedural values such as accountability, transparency and needs-based approaches. However, some authors challenge the applicability of values such as neutrality or impartiality^16^^,^ 17, while other values are considered as “tools” to carry out the operations. Terry^18^ refers to the contested principle of neutrality, stating that in the Afghan context, “neutrality was still an appropriate means to gain access to people in need.” On the other hand, values such as solidarity, defending human rights, or accountability are much less contested. However, as soon as values conflict with each other, humanitarian aid workers are likely to face a dilemma. Therefore, the proposed framework aims to raise awareness that certain issues faced by humanitarian aid workers are ethical issues, as opposed to geopolitical or managerial questions, and humanitarian actors are therefore likely to face dilemmas that might lead to moral distress. This is particularly applicable to professionals who should adhere to particular codes of ethics, such as physicians. It also suggests that certain values can be attributed to the different levels of decision-making, namely at the macro-, meso-, and micro-level (Table 1).

Second, a *procedural process* is proposed providing a ten-step approach (Table 2) is proposed to ensure that ethical considerations are factored into the decision-making process and addressed in an adequate manner. This model provides a structure and promotes transparency of the decision-making process, which allows for a comprehensive monitoring and evaluation of humanitarian programs. Third, the framework formulates *institutional requirements*, drawing parallels to a tool that has proven useful in the clinical setting. Overall, the proposed framework has been based on the experiences of several humanitarian fieldworkers and was tested and deemed useful by humanitarian aid workers and donor agencies. This approach was designed so that it can evolve further and be adjusted to specific needs in the field.

**Part One: Defining a set of values guiding humanitarian action**

The values listed in Table 1 have been drawn from an empirical study by one of the authors that analyzed the value statements of 46 international organizations active in the humanitarian context^25^. The ten most frequently mentioned values by the humanitarian organizations were: health, independence, poverty reduction, accountability, humanity, sustainability, transparency, relief, dignity, and empowerment.

**Table 1: Set of values commonly shared among humanitarian aid organizations:** 

	Specific values		Cross-cutting values	
	Substantive	Procedural	Substantive	Procedural
Macro and meso level (Headquarter and regional level)	Poverty reduction Sustainability	Inclusiveness Accountability Reasonableness Critical analysis	Duty to provide care Vulnerability Justice Solidarity Equity Beneficence Non-maleficence Cost-effectiveness Stewardship	Transparency Scrutiny
Micro level (Local level)	Focus on the worst off Self-determination Security Autonomy Beneficiary-centeredness Non-discrimination Empowerment	Responsiveness Engagement Timeliness Protecting confidentiality Effectiveness Efficiency		

These values^[4]^ represent only a selection that can serve as a starting point for individual organizations developing their own value matrix. These organizations will find that some values are specific to levels relevant to their decision-making structure, while others cut across different issues. For instance, aid workers at the micro-level might not be expected to worry about the sustainability of organizations, which may be of more concern to headquarters. However, the aid workers might be expected to deliver aid in a non-discriminatory way. Some of the values in an organization’s value matrix will be more substantive, while others are more procedural. Although the classification of some values can be contested, the point is to revisit two central questions organizations should ask themselves: a) What are the moral values we try to realize with our work, and b) How do we want to obtain these goals?

**Part Two: A ten-step approach to ethical decision-making in humanitarian aid**

Based on the assumption that decision-makers are aware of the need to a) identify ethical issues in the humanitarian context as such, and that b) the organization embraces values, norms, or principles that can serve to justify a response, we suggest a ten-step approach that supports aid workers at macro-, meso- and micro-levels alike in their difficult task of making ethically sound decisions. This approach is based on tools that are well-established and effective in clinical settings.

However, before initiating the first step, the following preconditions should be met. First, a platform to discuss needs should be made available where opinions can be shared openly. This might entail a round-table discussion similar to ethical consultations in a clinical setting. These discussions would ideally evolve into regular meetings of an established group of ethics committees. Second, all participants acknowledge that various interests may affect the decision-making process, such as those of organizations themselves, the donor community, local or regional governments, and others. It is, however, paramount that the interests of the beneficiary are at the center of the debate during the discussion. Finally, an ethicist should oversee the discussion, ensuring that all participants have equal opportunity to express their viewpoints. Based on the success of this approach for dealing with ethical dilemmas in clinical settings, we believe that a formally trained ethicist would be a valuable asset to humanitarian organizations. Ethicists have a unique ability to identify critical ethical issues and act as an intermediary between multiple parties in order to resolve them fairly. Hiring such a professional would not require significant financial investment from an organization and might also provide aid workers, managers, or physicians with some degree of formal ethics training.

**Table 2: A ten-step approach to ethical decision-making in humanitarian aid:** 

	Activity	Description of the activity
1	Gathering evidence	Situation analysis involving all concerned actors Define who is part of the decision-making process and justify the inclusion/exclusion of stakeholders Decide who is in charge of the process (in absence of an ethicist)
2	Specifying values, norms or principles	Description of the key values at stake - Individual values and interests - Institutional values and interests - Legal norms - Social norms
3	Critical examination of arguments	Critical examination of the legal, factual and ethical issues Identify the ethical conflict precisely
4	Defining options	Describe options and their potential and forecasted consequences on each actor
5	Weighing	Weighing of options in the light of the identified arguments and available evidence
6	Elaborating decisions	Deciding on the most appropriate option
7	Providing justification	Justifying the choice of the option Ensure transparency of the choice including reasons and justifications Define limits of the decisions
8	Implementation	Define measures to improve successful implementation Define indicators to monitor progress
9	Monitoring and evaluation	Monitoring of indicators assessing the decision's impact Providing evidence of the decision's impact on all concerned (internal/ external stakeholders) Generating evidence of benefits and harms Final evaluation of the decision-making process
10	Recommendations	Recommendations for future actions

This proposed approach embodies the core of the ethical framework by providing a structure to the decision-making process by humanitarian actors. It improves its transparency, which is paramount to promoting justice. This ten-step approach is applicable at a macro-, meso- and micro-level.

**Means of verification**

To test whether the **decision was correct, the following means of verification are considered helpful: a) decisions are based on available evidence^19^, b) decision-makers would agree with the defined option if they were in the beneficiaries' situation and c) decisions are relevant and allow for revision and appeals^20^.

A well-documented and transparent decision-making process is essential to permitting review and questioning whether or not the decision was appropriate. As such, organizations can monitor and evaluate the operational implementation of the program and retrospectively assess the portrayed options and whether another decision would have been a better one. These factors thereby contribute to the continuous improvement of decision-making processes within the organization.

**Part Three: Institutional requirements for high ethical standards in humanitarian aid organizations**

Guiding humanitarian aid workers in their difficult task to make decisions and allocate resources^21^, requires skilled professionals especially in challenging contexts. In many situations, staff will likely find it difficult to a) identify issues as being ethical ones, b) shape decision-making processes in a way that complies with the set of procedural values embraced by an organization, and c) help formulate core arguments and analyze them in light of relevant empirical information.

In clinical ethics, the hub and spokes model^8^ has proven to be quite useful. A trained ethicist acts as the “hub,” representing the organization’s central resource for ethical issues. The ethicist may help define an organization’s value matrix, develop the structures and processes required for the ten-step approach to ethical decision-making, and run an ethics consultation service. The ethicist will also actively involve interested colleagues in ethics activities, offer continuous educational opportunities, and create the focal point or “spokes” for ethical issues at various levels and in the different departments of the organization. In this way, ethical expertise is disseminated but not diluted.

**A model for institutional ethics resources (modified from MacRae et al., 2005):**
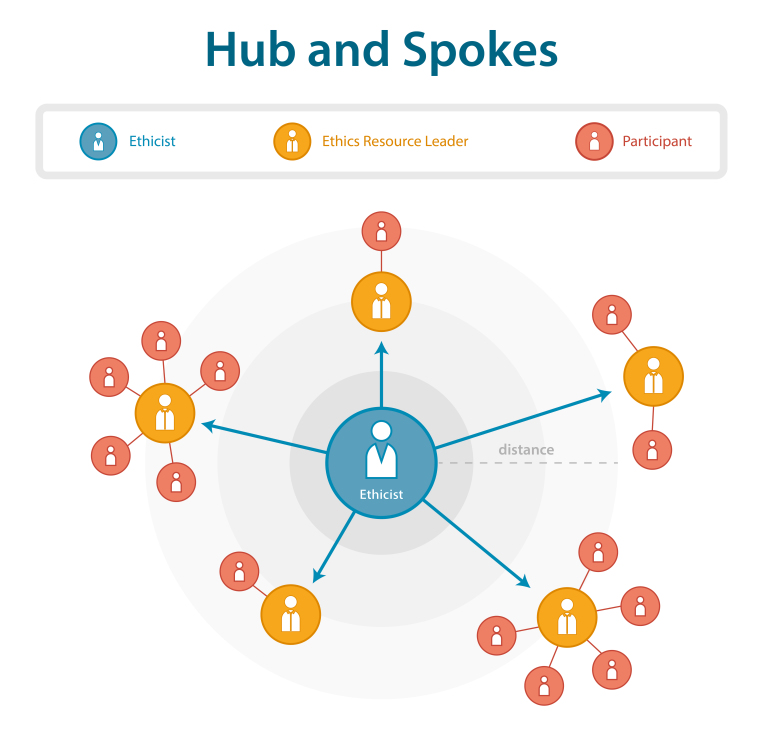

As with clinical practice, humanitarian contexts are often highly challenging and ideally require a fully trained ethicist to assist and act as a mediator who guides the decision-making process. The experienced ethicists train all ethics resource leaders to ensure that skilled humanitarian aid workers are available and able to act as ethicists at all levels. Depending on the level at which ethical consultation is needed, either the experienced ethicists or the ethics resource leader constitute a committee including key stakeholders as participants. Subsequently, the committee either decides to proceed according to the principles stated in the ethical framework proposed here or agrees to a set of core principles for future reference and to shape the decision-making process.

## Remaining challenges

Looking across humanitarian organizations show that a wide range of values continues to prevail, and reaching a consensus on a set of core values or principles is a challenging task. Yet, humanitarian action is embedded in substantive and procedural values of ethical relevance. At present, however, identifying conflicting values is not part of many organizations’ decision-making culture, which is predominantly marked by a geopolitical discourse. Therefore, a platform is required to allow for such debates.

Beginning with the framework described in this paper, each organization must elaborate their own approach to ethical decision-making, attuned to their value set and the ethics resources available at their institution. Importantly, regardless of the detail in the structures and processes, they must be conceived as a learning system that begins imperfectly but aims at continuous improvement. In addition to tailoring the overall approach to the specific needs and features of individual humanitarian aid organizations, a further challenge will be creating opportunities for exchange between organizations. It will be important for organizations to benefit mutually from experiences and explore potential for common standards that might emerge.

Another anticipated obstacle involves the issue of resources required for establishing or upscaling an ethics structure in humanitarian aid organizations. With regard to this concern, it is important to consider that to pursue a greater goal, material and human resources are at risk of being misused or instrumentalized. The aim of the ethical framework is to raise such concerns and demand specific justifications in such cases. Solid, ethical decision-making processes can help save resources by adding a level of scrutiny that may help avoid bad or wasteful choices. The meso-level case described above provides a useful example in which this approach would have been beneficial. The organization's primary goal in distributing food aid was not to improve the local population’s nutritional status, but, instead it was to increase access to local leaders. Therefore, ethicists play an important role in guiding organizations through the decision-making process. It is left up to each organization to find the mechanism that best suits their decision-making process. Applying the hub and spokes model does not require extensive additional human resources, but will require staff with a specific profile who already have the necessary competencies.

## Conclusion

In this paper, we emphasize that ethical issues are inherent in humanitarian action. We highlight sp,e possible ethical dilemmas and decisions faced by humanitarian workers and show that their participation in the decision-making process was often lacking. The proposed ethical framework strives to a) stress certain values that guide humanitarian action, b) recommend a ten-step approach that strengthens humanitarian aid workers’ capacity to identify and address ethical issues when making decisions, and c) suggest a model (“hub and spokes”) that helps define the organizational resources needed to implement the framework.

The ethical framework aims to support humanitarian aid workers and other stakeholders in their decision-making process, which will ultimately improve the processes of project cycle management. This is especially relevant because humanitarian action remains a challenging field involving multiple actors that adhere to different sets of principles in often highly complex and volatile environments. The proposed tools also foster exchange through mediated discussions involving all stakeholders, which in turn promotes coherence in decisions and outcomes.

If humanitarian organizations had adopted such an approach in the sample cases presented in this paper, the outcome would have been significantly different. For example, repeated budget allocations would not occur without involving the program managers in the field, who are directly concerned with such decisions. Material resources would not be used to meet covert goals and staff members would be educated so that they can provide informed consent prior to their deployment. Moreover, measures would be taken to mitigate the harm of any operational program on the beneficiaries.

Because humanitarian action frequently evolves in highly volatile contexts, promoting ethical considerations within and across actors and organizations is essential to avoid exacerbating the challenges faced by victims of disasters.

## Footnotes

[1] The macro-, meso- and micro-levels chosen in this context represent the three main levels of decision-making within a humanitarian organization, partially excluding a social ecological^27^.

[2] Beauchamp and Childress (2009: 23) propose six conditions that ‘must be met to justify infringing one prima facie norm to adhere to another. According to the authors 1) good reasons can be offered to act on the overriding norm rather than on the infringed norm. 2) The moral objective justifying the infringement has a realistic prospect of achievement. 3) No morally preferable alternative actions are available. 4) The lowest level of infringement, commensurate with achieving the primary goal of the action, has been selected. 5) Any negative effects of the infringement have been minimized. 6) All affected parties have been treated impartially.

[3]These tools are described in Fletcher’s Introduction to Clinical Ethics 13, Beauchamp and Childress, Principles of Biomedical Ethics^22^, Singer^23^, Bioethics at the bedside: a clinicians guide, Wall^24^, Ethics for International Medicine: A Practical Guide for Aid Workers in Developing Countries.

[4] For the purposes of this paper, we do not distinguish between values, norms and principles. The values in the table could also be phrased as principles or norms.
